# What is known about what works in community-involved decision-making relating to urban green and blue spaces? A realist review protocol

**DOI:** 10.1186/s13643-023-02333-y

**Published:** 2023-09-20

**Authors:** Emmylou Rahtz, Sarah L. Bell, Alexander Nurse, Benedict W. Wheeler, Cornelia Guell, Lewis R. Elliott, Catharine Ward Thompson, Craig W. McDougall, Rebecca Lovell

**Affiliations:** 1 European Centre for Environment and Human Health, University of Exeter Medical School, Peter Lanyon Building 12, Penryn, Cornwall, TR10 9FE UK; 2 Department of Geography and Planning, Roxby Building, University of Liverpool, Liverpool, L69 7ZT UK; 3 OPENspace Research Centre, University of Edinburgh, 74 Lauriston Place, Edinburgh, EH3 9DF UK

## Abstract

**Background:**

There is now a relatively well-established evidence base suggesting that greener living environments and time spent in urban green and blue spaces (UGBS) can be beneficial for human health and wellbeing. However, benefits are not universal and there remain widespread social inequalities in access to such resources and experiences, particularly along axes of class, race, ethnicity, age and disability, and in relation to efforts to increase the availability and accessibility of such spaces. These injustices often relate to distributive, procedural and recognition-based processes. There is growing interest in how to ensure that efforts to increase access to or use of UGBS (whether through infrastructural or social programmes) result in equitable outcomes whilst minimising potential for exacerbating existing inequalities and injustices. Community engagement is considered an important step towards more inclusive UGBS decision-making, from planning and design to management and maintenance processes. It is thought to contribute to better and more widely trusted decisions, enhanced democracy, community satisfaction, civic interest and feelings of green space ownership, and greater longevity of UGBS projects. However, uneven representation and barriers to participation can create imbalances and undermine these benefits.

**Methods:**

An iterative, multi-stage realist-inspired review will be conducted to ask what works, in what context and in what ways relating to the meaningful involvement of communities in UGBS decision-making, focusing on the skills, capacities and capabilities of different stakeholders and the role of contexts and processes. ‘Effectiveness’ (or what works) will be understood as a multifaceted outcome, encompassing both the processes and results of community engagement efforts.

Following a scoping stage to identify initial programme theory, inclusion/exclusion criteria and derive search terms, relevant databases and grey literature will be searched to identify interdisciplinary literature in two phases. The first phase will be used to further develop programme theories, which will be articulated as ‘if then’ statements. The second phase searches will be used to identify sources to further explore and evidence the programme and formal theory. We will assess all includable evidence for conceptual richness, prioritising more conceptually rich sources if needed.

**Discussion:**

The realist synthesis will explore the key context, mechanism and outcome configurations that appear to explain if and how different approaches to community-involved UGBS decision-making are or are not effective. We will consider factors such as different conceptualisations of community, and if and how they have been involved in UGBS decision-making; the types of tools and approaches used; and the socio-cultural and political or governance structures within which decision-making takes place.

## Background

There is now a relatively well-established evidence base suggesting that greener living environments and time spent in urban green and blue spaces (UGBS)—for example, parks, gardens, woodlands, trails, canals, quaysides, riverbanks and coastal settings—can be beneficial for human health and wellbeing [1]. These resources and place encounters are thought to promote opportunities for physical activity [2], social interaction [3], and enhanced mental health through stress reduction and cognitive restoration [4]. However, benefits are not universal and there remain widespread social inequalities in access to such resources and experiences, particularly along axes of class, race, ethnicity, age and disability [5], and in relation to efforts to increase availability and accessibility of such spaces.

Inequalities in UGBS access and use have gained particular attention within the wider body of environmental justice scholarship [6]. The environmental justice movement began in the USA largely in recognition that communities subjected to race and class-related inequalities tended to be at risk of greater exposure to pathogenic components of the environment [7]. Over the last 10 years or so, studies have also begun to explore differential access to potentially salutogenic environmental resources [8, 9], including urban greenspace. These studies have identified mixed and often contradictory findings concerning the proximity of socio-economically disadvantaged groups to greenspace and ‘green’ neighbourhoods [10–12].

Environmental justice scholars typically conceptualise justice as trivalent, namely distributive, procedural and recognition-based justice [6]. In the context of UGBS, these dimensions include consideration of how the environmental benefits of UGBS are spatially and socially distributed or allocated (distributive), who can participate meaningfully in UGBS decision-making processes (procedural), and the extent to which the distinct UGBS values, identities and preferences of specific social groups are respected and attended to in these processes (recognition). A failure to attend to such dimensions within UGBS interventions can lead to detrimental processes of green (or ‘ecological’) gentrification and displacement, with minority and low-income residents witnessing the ‘greening and rebranding’ [13] of—and subsequent exclusion from—their communities for the benefit of ‘socially and racially privileged residents’ [13]. Such projects have been linked to ‘green locally unwanted land uses’ (GreenLULUs) in historically distressed neighbourhoods, disproportionately affecting or displacing the most economically marginalised community members [9].

More recently, Anguelovski et al. [13] have also called for (a) reparative or restorative justice lenses to assess the extent to which UGBS interventions ‘openly acknowledge and address histories and geographies of oppression and exclusion’; (b) preventive justice to identify UGBS practices that could prevent or reduce the likelihood of future harm; (c) hermeneutical justice, ensuring marginalised social groups have the discursive and material tools to reflect on and share their distinctive experiences and perspectives regarding UGBS interventions; and (d) epistemic justice, revealing and challenging prejudice from listeners that otherwise undermines the credibility of social groups contributing such perspectives.

Community engagement is considered an important step towards more inclusive UGBS decision-making, from planning and design to management and maintenance processes [6, 14]. ‘Community’ typically refers to both communities of place (where participation is shaped by shared locality) and identity (where inclusion is defined by individual or collective characteristics) and communities of interest (with participation shaped by shared interests) [15]. However, there have been calls within the wider public health literature for more critical attention to the concept of community, how it is operationalised within public health interventions, and how socio-spatial boundaries to community are negotiated and challenged [16]. Similarly, there is a need to critically explore conceptualisations and processes of community ‘engagement’. Engagement has often been characterised hierarchically, as typified by Arnstein’s ladder [17], ranging from non-participation and education of the community on the lower rungs through to citizen control at the top of the ladder. However, there have been successive moves away from value-laden models, recognising that more engaged processes may not be most appropriate in all situations. More recent models including democracy cubes [18], wheels [19] and trees of participation [20] seek to acknowledge power dynamics and the influence of context.

Community engagement in UGBS interventions is thought to contribute to better and more widely trusted decisions, enhanced democracy, community satisfaction, civic interest and feelings of green space ownership, and greater longevity of UGBS projects [21–23]. However, unbalanced representation and barriers to long-term participation can create inequalities and undermine these benefits [24]. This is a particular risk when community engagement is under-resourced and conducted primarily as an ‘after thought’, when the loudest voices are prioritised over those of people who are already most excluded and when greenspace and wider local and national governance structures are not conducive to genuine power sharing between governmental and non-governmental participants in the process [21].

There is a need to identify what forms of UGBS decision-making meaningfully engage with, reflect the needs and desires of and respond to the capacities of relevant communities. Community-based UGBS decision-making is complex and encompasses a variety of different methodologies (see Table 1) and tools. Tools used include informing methods such as PR (public relations) campaigns and lectures; methods that invite typically limited responses such as canvassing, public meetings, surveys or notification and comment; more consultative approaches including interviews, focus groups or facilitated group interactions, and expert workshops [25]; and those that enable co-creation such as learning alliances and living labs, which may combine several other methods [26]. Surveys and questionnaires have been the most widely used, and there is increasing use of social media and GIS-based approaches [27], although digital approaches can entrench exclusionary behaviour. The choice of approaches is driven by factors such as the specific motivations and drivers of the engagement, whether procedural (e.g. it is a statutory duty) or attitudinal (e.g. it is ‘right’ to hear the perspectives of local communities), as well as a variety of other considerations such as the resources available to support the engagement, and timing.

**Table 1:** Different dimensions of community-involved UGBS decision-making (derived from Concannon et al. [28]; Ferreira et al. [27]; Kliskey et al. [29])

**Driver:** A statutory duty; a step of an established design process; a desire to be inclusive; to create more trust in a decision-making process
**Intention:** Inform a decision, collaborative governance, collaborative management
**Resources:** Budget, time, tools, skills
**Actors:** National state actors, local authorities and government, civil and third sector groups, private sector, communities, individuals, and others
**Directionality:** Unidirectional (e.g. an information gathering or imparting exercise), or bi-directional (e.g. information is provided and gathered); top down, bottom up or partnership
**Timing and longevity:** The stage at which the community is involved, whether before the decision-making process has begun or during the process, and whether there is ongoing involvement and management
**Scale:** Relating to the extent and comprehensiveness of the engagement, ranging from a single information dissemination event to ongoing co-production
**Geography**: National, regional, local authority, neighbourhood/parish
**Roles and responsibilities:** Agency and accountability of different actors
**Accountability:** Legal (in accordance with local codes and frameworks), to funders/investors, to community
**Agency:** Level of influence e.g. are communities enabled to contribute to decision-making about meaningful, substantive issues or only trivial ones
**Commitments to act:** Benefitting the community, the ecosystem

Clarifying and understanding the methods, tools and resources needed for communities to be equitably included in such processes could help reduce the potential of unintended consequences of UGBS projects. To best understand what works, we need to explore *how* different approaches work, *where* and for *whom*. A realist review methodology is well-suited to understand ‘what works’ in community-engaged decision-making [30].

Realist reviews seek to reveal the interactive layers of how, why and for whom programmes achieve their observable outcomes. Such approaches recognise and respond to the complexity of these processes and seek to understand the chains of mechanisms that can lead to outcomes. Importantly, realist approaches can explicitly take account of the socio-political, physical and temporal context of the action. The resources and reasons are responsive to the conditions in which the individual, community or organisation is acting. This methodological perspective fits well with theoretical conceptualisations of how more meaningful involvement of people in decision-making might be enabled, focusing on the skills, capacities and capabilities of different stakeholders and the role of context rather than discrete actions which do not take such factors into account.

While there have been previous reviews which relate to this topic (for example, Ferreira et al. [27]), no realist synthesis, exploring if and how efforts to engage communities in UGBS decision-making are effective, was found. The review contributes to the Prevention Research Partnership-funded GroundsWell research programme (MR/V049704/1). GroundsWell takes a systems approach to understanding the multiple and interconnecting components of policy-making, practice, perception and people which together affect the presence, location, character and use of UGBS.

### Review objectives

In this realist theory-driven review, we will draw on a broad range of interdisciplinary literature to understand what is known about what works, for whom and in what contexts in community-involved UGBS decision-making, particularly where there is an ambition or likely impact of the use or change to the UGBS relating to human health. We will explore the key mechanisms that appear to explain if and how different approaches to community-involved UGBS decision-making are, or are not, effective. The overarching program theory we will test is that: In a context where infrastructural changes are taking place in urban settings that involve UGBS and affect communities (particularly marginalised and structurally disempowered communities), appropriate and sufficient consultation and engagement activities will ensure that communities’ needs are better understood and better implemented within plans.

To approach this, we will test the initial program theories shown in Table 2.

**Table 2:** High-level initial program theories

Level	Context	Mechanism	Outcome
**Infrastructure**	Funding for engagement is insufficient and piecemeal	Funding is guaranteed/ensured for the long term/continuous, giving professionals time to plan and engage	Sufficient resources for engagement and longer time frames mean more people can be engaged with (depth/breadth) and involved in decision-making
	Professionals do not have the tools they need to engage with community members	Contextually relevant, accessible and usable tools are made available	Appropriate tools enable engagement with and involvement of community members
**Institutional**	Engagement activities happen at the ‘wrong’ time meaning communities cannot meaningfully contribute to decision-making	Engagement activities are planned for an appropriate stage and with ample time	Planning allows communities to engage thoroughly throughout the process
	Existing decision-making processes do not require community members’ perceptions and expectations to be considered and integrated	Policy changes specify that community members’ perceptions and expectations must be considered and integrated into the decision-making process	Institutions factor in community engagement in order to abide by policies
	There is a lack of mandates to require meaningful depth (enough people in an individual community) and breadth (enough different types of communities/individuals) of engagement in decision-making	The mandates to meaningfully ensure depth and breadth are strengthened	Sufficient depth and breadth of community members are engaged with and involved in the decision-making process
**Inter-institutional**	Different bodies are unsure/unclear about how engagement should be initiated, and by whom	Systems are mapped to clarify structures and relationships, roles and responsibilities are clarified	Each organisation understands its role in the engagement process, and when action needs to be taken and by whom
	Local social structures and communities are fragmented	Mapping and networking activities improve cohesiveness	Local social structures and communities are better connected, facilitating engagement and collaboration
**Individual—professionals**	Professionals feel uncertain (e.g. lacking skills, knowledge and aptitude) about engaging community members	Professionals have access to training and resources that support their professional practice	Professionals feel more confident and able to engage with communities
	Professionals feel sceptical about engaging community members	Professionals are provided with evidence and case studies highlighting the benefits of community-involved decision-making	Professionals feel greater motivation to engage with community members
**Individual—community members**	Community members are sceptical about engaging with professionals and the decision-making process and have a sense of well-informed futility	Efforts are made to increase transparency and clarity about the decision-making process, to address community members’ doubts and redress previous negative experiences	Community members are reassured about the equity and validity of the process and are motivated to engage
	Community members feel unable or *are* unable to join the decision-making process and are therefore excluded from the process	More inclusive and diversified ways of engaging with communities, that take account of people’s time/capacity, are identified and utilised	A wider range of community members engage due to barriers to participation being reduced/removed
	There are power disparities within communities meaning that not everyone gets to/feels able to be involved	Equitable means of facilitating and encouraging participation which address power imbalances are used	More members of the community/s are engaged and get involved; the decision-making process itself fosters working relationships among the community
	Community members feel disempowered and lack confidence or motivation to be involved	A publicity drive through appropriate channels communicates the benefits of involvement	Community members enjoy the experience and feel empowered to become more involved in decision-making in future, with positive ripple out effects beyond the immediate programme and community

Within this process, we will consider a number of important sub-questions, including:

1. How is ‘community’ conceptualised and approached within UGBS decision-making and governance literature?
2. Which types of communities have been involved in UGBS governance, and at what stages/spatial scales of decision-making?
3. What tools and approaches have been used to engage communities in UGBS decision-making?
4. To what extent and in what ways have community members been excluded from such processes as a result of social inequalities pertaining to race, ethnicity, gender, sexuality, class, age and disability?
5. What types of governance structure(s) facilitate just and equitable community participation in UGBS decision-making?

Different forms of community-involved UGBS decision-making are characterised by a variety of different factors such as the drivers and intentions of the action, its timing, and methods of engagement (see Table 1). We define ‘community-involved UGBS (urban green/blue space) decision-making’ as approaches which seek explicitly to understand, include, integrate and/or act on the attitudes, perceptions, needs and desires of the people and communities who will be affected by the action being considered. Different forms of community-involved UGBS decision-making are characterised by a variety of different factors.

Furthermore, we understand ‘just and equitable community participation in UGBS decision-making’ to reflect parity of participation, both in terms of how situations/problems are framed and how decisions are made [31], including the *redistribution* of opportunities and means for all community members to participate fully, safely and to exercise genuine agency in the process; the *recognition* of social status inequalities, including efforts to address the misrecognition of individuals and community groups that have previously been rendered invisible or less integral to decision-making processes; and *representation*, broadening the meanings and types of tacit and embodied knowledge that are valued and the ways in which participation channels are framed and structured [32].

We will consider ‘effectiveness’ as a multifaceted concept in this context, encompassing both the processes and outcomes of community engagement efforts, rather than seeking a single outcome measure. Underpinning our understanding of effectiveness are the core dimensions of social justice described above, considering whether engagement processes have:

- Improved the equitable *distribution* of accessible UGBS across the community
- Enhanced opportunities amongst diverse community members and groups to use such settings in ways that are personally meaningful/relevant
- Increased the potential for local UGBS use to promote health and wellbeing amongst diverse community members
- Broadened who can participate fully in the varied processes (*procedures*) involved in UGBS decision-making, ensuring people have the resources and tools to share their perspectives and knowledge *and* to effect meaningful change through their participation
- Respected and attended to—i.e. *recognised*—the distinct UGBS values, identities and preferences of specific social groups in these processes
- Confronted power imbalances and sought to address problematic histories of oppression or exclusion of specific community groups in such decision-making processes;
- Sensitively managed tensions arising in the process in ways that do not undermine the credibility or agency of those who participate.

Determining effectiveness, or success, is not necessarily simple. In many cases, UGBS decision-making may result in negative outcomes for some groups (e.g. the loss of trees to make way for an accessible path); however, it could be that the action was considered to have been ‘effective’ as different perspectives were heard and considered in the making of the decision. These understandings and definitions will be revisited and revised in light of the literature.

The review will focus primarily on decision-making processes in the United Kingdom (UK). This somewhat narrow geographical focus is justified by the particular socio-cultural, and the political and procedural contexts within which such decision-making happens.

## Methods

An iterative, multistage realist review methodology will be used [30, 33–36]. The review will be informed and guided by stakeholders (as discussed further below).

### Refining the scope of the review and identifying initial theory

First, the team will further refine and define the scope of the review. Key concepts, definitions and theories in the disparate literature will be identified. Initial understanding of the ‘bounds’ of the action (e.g. what does and does not ‘count’ as community-involved UGBS decision-making) will be clarified. This will relate to articulating the key components of the actions, the outcomes they aim to achieve and how the components of the action relate to the outcomes (the mechanisms of action).

Examples of primary literature detailing evaluations and descriptions of community-involved UGBS decision-making will be collated. This will guide the identification of key search terms as well as clarify the size of the available literature, thus informing whether or not steps such as prioritisation of sources according to conceptual richness will need to be adopted.

The identified concepts and theories will be used to further develop a conceptual model of how community-involved UGBS decision-making works and to articulate the specific programme theories—realist explanations of how a set of actions leads to outcomes—to be examined in the review. This process will be supported by the stakeholder group.

### Stakeholder group

A stakeholder group of relevant professionals (e.g. those involved in the management and governance of UGBS) and communities and individuals will be convened. The stakeholder group will:

- Suggest theories of how community-based UGBS decision-making works and inform the conceptual model
- Provide examples of community-based UGBS decision-making
- Suggest relevant source of literature on community-based UGBS decision-making
- Help prioritise, if needed, the focus of the review and literature used

We will also draw on principles from Bell and Reed [20].

### Searches

As is common for realist reviews, a wide range of literature and resources that relate to the programme theories will be sought [30, 33, 34]. The searches will be conducted in two key phases:

#### First phase

The initial searches will gather relevant material to explore how community-involved UGBS decision-making is thought to work and further develop the programme theories. The literature will be synthesised (see the ‘Analysis and synthesis’ section for details), and theories will be articulated as ‘if then’ statements. We will consider programme components and contexts, such as the different dimensions of community-involved UGBS decision-making (see Table 1), in interpreting the evidence [27–29].

Search terms for the first phase will be derived from the literature identified in the scoping stage and will likely relate to the focus of actions (e.g. green/blue infrastructure, greenspace/bluespace, parks); the types of engaged decision-making (e.g. shared governance; methods of engaged decision-making (e.g. consultation, community forums); and potentially the communities of interest (e.g. using terms such as deprived, marginalised, underserved, structurally disempowered).

#### Second phase

In the second phase, more targeted searches will be conducted to further explore and evidence key programme and formal theories.

Search terms for the second phase will be derived from the key topics to be further explored.

For both phases, it is likely that relevant literature will be widely dispersed and found across academic disciplines (e.g. public health, social sciences, planning) and non-academic sectors (such as strategy documents and project reports). As such, searches will focus on both academic databases and sources of grey literature. The academic databases we will search will include ASSIA, MEDLINE, Embase, Web of Science, Scopus, GreenFILE and PsychInfo. Grey literature will be found through Google searches, contact with key informants (including the stakeholder group, and key authors) and searching relevant institutional libraries and websites. Forward and backward citation searches will also be used.

Results of the searches will be recorded using the Preferred Reporting Items for Systematic Reviews and Meta-Analyses (PRISMA) tool [37].

### Study selection (inclusion/exclusion PICOS)

Study selection will follow the typical methods. One team member will review titles and abstracts. Study selection at full text will be undertaken by two team members. Disagreements relating to inclusion will be resolved through discussion and referral to a third team member if necessary.

Following the guidance of Rycroft-Malone et al. [34], initial study selection at title and abstract will be more inclusive than may be traditional for systematic reviews to allow for the consideration of diverse forms of evidence and the plurality of practices and language used around the topics of community-based UGBS decision-making. Studies will be considered on the criteria of relevance—their contribution to our process of theory building—and rigour—the credibility and trustworthiness of the methods used [38]. A final decision on whether or not individual sources of evidence are includable will be determined by considering the question posed by Rycroft-Malone et al. [34], ‘Is the evidence provided in this theory area good and relevant enough to be included in the synthesis?’. The stakeholder group will be invited to help further refine these criteria.

The evidence identified through the first and second phases of searches will be judged against the following criteria:

#### Study type inclusion criteria

We will take an inclusive approach to the types of evidence incorporated in both phases. Evidence will be assessed on its potential to contribute to theory development rather than according to factors such as the ‘robustness’ of the design. Therefore, we will seek quantitative, qualitative or mixed-method academic literature such as primary studies, process evaluations, evidence syntheses and review articles, as well as editorials and commentaries. From the grey literature, we will seek policy and strategy documents, quantitative or qualitative project and procedural evaluations, and commentaries.

#### Population inclusion criteria

In both phases, we will include evidence relating to populations or communities involved or invited to be involved in UGBS decision-making. Priority will be given to identifying and including evidence which relates to populations or communities that are or have been subject to socio-cultural and/or political processes of exclusion or marginalisation. Evidence relating to the UK will be prioritised; however, that relating to other contexts may be included judiciously where there are gaps in understanding or a paucity of evidence relating to the UK contexts.

#### Intervention (or ‘action/approach’) inclusion criteria

In the first phase, we will include evidence relating to community-involved UGBS decision-making interventions, actions or approaches as defined above. Examples are included in Table 1; however, the bounds of this will be finalised in the initial scoping stage and will inform these inclusion/exclusion criteria.

In the second phase of searches, inclusion will relate to interventions, actions and approaches that can further clarify mechanisms of action. It is likely that this evidence will be drawn from a wider literature and will not necessarily be directly related to community-involved UGBS decision-making. For example, evidence relating to community engagement in regard to more broad public health actions may be selected if it could elucidate processes such as modes of engagement.

#### Comparators

Due to the nature of the topic, it is considered unlikely that any evidence produced using controlled study designs will be identified. No comparator criteria will be used to assess the evidence for inclusion.

#### Outcomes

As noted previously, the outcomes of efforts to undertake community-based UGBS decision-making could have a range of potential outcomes. We expect that the outcomes assessed in the sources we include will relate to the factors that have been identified as success concepts (distribution of resources; participation in procedures; recognition; addressing power imbalances, oppression or exclusion; and management of tensions) such as:

- Measures or reports of community awareness of UGBS action
- Measures or reports of community involvement in priority setting for UGBS action
- Measures or reports of community involvement in design of UGBS action
- Measures or reports of community involvement in governance, management or monitoring of UGBS
- Measures or reports of attitudes towards engagement processes at any stage of UGBS action
- Impact assessments of UGBS on communities where engagement took place

Duplicates will be identified and removed.

### Quality assessment

First, we will assess all includable evidence for the criteria of relevance and rigour [38] and conceptual richness. We will follow the approach taken by Pearson et al. [39], which built on work by Ritzer [40] and Roen et al. [41]. This approach aims to classify evidence as to whether it is ‘conceptually rich’, ‘not conceptually rich but has thicker description’ or only has ‘thinner description’. If there is more evidence than can be reasonably dealt with in this study, we will use the results of the classification to prioritise the ‘conceptually rich’ and ‘not conceptually rich but has thicker description’ sources.

Where appropriate, we will apply standard quality assessment tools to the evidence we include in the review. If any randomised control trials (RCTs) are identified, we will use the Cochrane Collaboration’s risk of bias tool [42]. For all other designs of primary study, we will use the Wallace tool [43].

### Data extraction

The results of the searches will be managed in a reference management system.

Following Pearson et al. [39], we will extract key information, into bespoke forms, on each piece of evidence relating to:

- *Evidence characteristics*: author, date, context, type of evidence (e.g. primary study or other)
- *Study methods (if relevant)*: methods, analytical approach, etc.
- *Evidence summary*: summary of outcomes (e.g. see the ‘Review objectives’ section above), summary of argument, strategy, etc.
- *Non-primary study evidence*: key explanatory concepts and explanations
- *Other information*: other factors not covered in the previous criteria.

We will not seek to extract exhaustive ‘results’ from the sources as this is impractical for this form of review. Instead, we will work with the original sources at this stage of the work. We will familiarise ourselves with the evidence through reading, discussion and note-taking. We will seek information which will inform our understanding of community-involved UGBS decision-making programme theory, searching for the factors which appeared related to the outcomes observed or intended. We will also look for information relating to the influence of programme or setting resources, and so on.

Data will be extracted by one team member, with a subsample checked by a second team member. At this stage, the evidence will be managed in NVIVO, with coding according to key themes.

### Analysis and synthesis

The aims of the synthesis are to explore the key context, mechanism and outcome configurations that appear to explain if and how different approaches to community-involved UGBS decision-making are or are not effective. We will consider factors such as different conceptualisations of community, and if and how they have been involved in UGBS decision-making; the types of tools and approaches used; and the socio-cultural and political or governance structures within which decision-making is taking place.

We will follow established approaches to synthesis [30, 33, 34]. First, the sources of evidence will be categorised according to the revised programme theory. It will then be tabulated to aid comparative approaches. Subsequent analytic steps we will take include juxtaposition of identified evidence, reconciling sources of evidence, consolidating the evidence and situating the evidence in context. Any identified limitations of each source of evidence will be considered throughout the analysis synthesis. We will not seek to include or exclude evidence according to the results of the risk of bias assessments; however, we will take the results into account when considering the relative explanatory value of each source of evidence.

Through the analysis, we will aim to articulate the contextual influences that are hypothesised in our programme theories to have triggered the relevant mechanism to generate the outcomes relating to community engagement in decision-making [33]. Additional formal theory (explanations of the underlying assumptions about how initiatives work, identified through the second phase searches) will be used to help explore linked processes. We will work with our stakeholders to validate the findings.

Results will be presented following the Realist And Meta-narrative Evidence Syntheses: Evolving Standards (RAMESES) publication guidelines [33].

## Data Availability

Data sharing is not applicable to this article as no datasets were generated or analysed during the current study.

## Abbreviations

UGBS: Urban green/blue space
PRISMA: Preferred Reporting Items for Systematic Reviews and Meta-Analyses
PICOS: Patient/population, intervention, comparison and outcome
RAMESES: Realist And MEta-narrative Evidence Syntheses: Evolving Standards
